# Electrospun Chitosan Functionalized with C12, C14 or C16 Tails for Blood-Contacting Medical Devices

**DOI:** 10.3390/gels8020113

**Published:** 2022-02-12

**Authors:** Monica Dettin, Martina Roso, Grazia M. L. Messina, Giovanna Iucci, Valentina Peluso, Teresa Russo, Annj Zamuner, Marta Santi, Sabrina Milan Manani, Monica Zanella, Chiara Battocchio, Giovanni Marletta, Michele Modesti, Mario Rassu, Massimo De Cal, Claudio Ronco

**Affiliations:** 1 Department of Industrial Engineering, University of Padova, Via Marzolo 9, 35131 Padova, Italy; monica.dettin@unipd.it (M.D.); martina.roso@unipd.it (M.R.); michele.modesti@unipd.it (M.M.); 2 Department of Chemistry, University of Catania, Viale Andrea Doria, 6, 95125 Catania, Italy; grmessi@unict.it (G.M.L.M.); gmarletta@unict.it (G.M.); 3 Department of Sciences, University Roma Tre, Via della Vasca Navale 79, 00146 Rome, Italy; giovanna.iucci@uniroma3.it (G.I.); marta.santi@uniroma3.it (M.S.); chiara.battocchio@uniroma3.it (C.B.); 4 Institute of Polymers, Composites and Biomaterials, National Research Council of Italy, Viale J.F. Kennedy 54−Mostra d’Oltremare PAD. 20, 80125 Naples, Italy; valentina.peluso@ipcb.cnr.it (V.P.); teresa.russo@unina.it (T.R.); 5 Department of Nephrology, Dialysis and Transplantation, International Renal Research Institute (IRRIV), AULSS8, San Bortolo Hospital, Viale Rodolfi 37, 36100 Vicenza, Italy; sabrina.milan@aulss8.veneto.it (S.M.M.); monica.zanella@aulss8.veneto.it (M.Z.); mario.rassu@aulss8.veneto.it (M.R.); massimo.decal@aulss8.veneto.it (M.D.C.); claudio.ronco@unipd.it (C.R.); 6 Department of Medicine (DIMED), University of Padova, Via Giustiniani 2, 35128 Padova, Italy

**Keywords:** functionalized chitosan, electrospun scaffolds, XPS, dynamic mechanical analysis, Gram+ and Gram− bacteria

## Abstract

Medical applications stimulate the need for materials with broad potential. Chitosan, the partially deacetylated derivative of chitin, offers many interesting characteristics, such as biocompatibility and chemical derivatization possibility. In the present study, porous scaffolds composed of electrospun interwoven nanometric fibers are produced using chitosan or chitosan functionalized with aliphatic chains of twelve, fourteen or sixteen methylene groups. The scaffolds were thoroughly characterized by SEM and XPS. The length of the aliphatic tail influenced the physico-chemical and dynamic mechanical properties of the functionalized chitosan. The electrospun membranes revealed no interaction of Gram+ or Gram− bacteria, resulting in neither antibacterial nor bactericidal, but constitutively sterile. The electrospun scaffolds demonstrated the absence of cytotoxicity, inflammation response, and eryptosis. These results open the door to their application for blood purification devices, hemodialysis membranes, and vascular grafts.

## 1. Introduction

Blood-contacting medical devices, such as vascular catheters, hemodialysis filters, hemopurifiers, blood vessel substitutes, vascular grafts, stents, and heart valves, are routinely used in healthcare settings [1,2]. Such devices should be biocompatible, hemocompatible, and resistant to surface-initiated blood coagulation processes and adverse immune reactions [3]. However, devices such as central venous catheters (CVCs) are associated with unacceptably high levels of treatment complications, including occlusion, thrombosis and infection. Nowadays, the gold standard for blood-contacting materials is Dacron or Teflon. Both synthetic and natural materials have been recently investigated in an attempt to reduce protein and cell adsorption usually favored by electrostatic and hydrophobic interactions between the adsorbed protein and the artificial surface: these materials include poly(ethylene oxide), pyrolytic carbon, albumin, phosphorylcholine, and elastin-inspired protein polymers [4]. Chitosan derives from the partial deacetylation of chitin, a natural biopolymer [5], and it is considered a biologically renewable, biodegradable, biocompatible and nonantigenic material [6]. The acetylation degree (DA) of chitosan is highly correlated to the biodegradation, biocompatibility and growth inhibitory effect on bacteria [7,8,9,10] and a deacetylation grade of around 70% resulted favorably for biomedical applications. The presence of free amino groups in the polymer backbone facilitates chitosan functionalization. In particular, the reaction with aldehydes and ketones allows anchoring molecules by the formation of Schiff bases that, upon reduction, ensures selective and stable covalent bonds [11]. A particularly favorable feature of chitosan is its antibacterial capacity [12]. The most prevalent proposed antibacterial activity of chitosan is by binding to the negatively charged bacterial cell wall causing disruption of the cell, thus altering membrane permeability, followed by attachment to DNA causing the inhibition of DNA replication and subsequently cell death [13]. The positive charged groups of chitosan are probably responsible of antibacterial properties (advantage), but also of protein adsorption (disadvantage). The necessity of reducing protein and cell adsorption drive us to explore the properties of chitosan modified by the addition of hydrocarburic tails (12, 14 or 16 methylene units). However, chitosan can be electrospun in order to obtain matrices with micro- and nano-metric structures for biomedical applications. Fibers composition, diameter, alignment and scaffold porosity can be tailored to the specific cell or tissue types [14,15] and the high area-to-volume ratio offers the possibility to improve surface decoration with bioactive molecules [16]. Furthermore, the tunability of all the previously cited physical properties of the electrospun chitosan matrices make them a promising candidate for the development of next generation membranes for hemodialysis [17].

**Figure 1:**
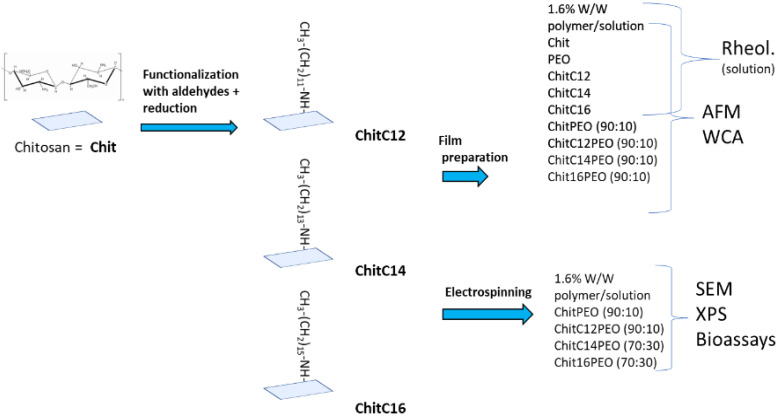
Schematic representation of the experimental plan with abbreviations.

The structure of our fibrous scaffolds (ChitC12, ChitC14 and ChitC16) are characterized by SEM. Surface composition is ascertained by XPS both for plain material and for electrospun scaffolds, whereas wettability (contact angle) is determined for functionalized chitosan films. Dynamic mechanical analysis (DMA) and AFM tests are performed to assess the viscoelastic and local material properties of the chitosan solutions and films, respectively. The biological characterization is carried out by evaluating the scaffolds’ interaction with renal tubular cells (RTCs), blood extracts or Gram+ and Gram− bacteria. A schematic representation of the complete experimental plane is reported in Figure 1.

## 2. Results and Discussion

### 2.1. SEM Analysis

**Figure 2:**
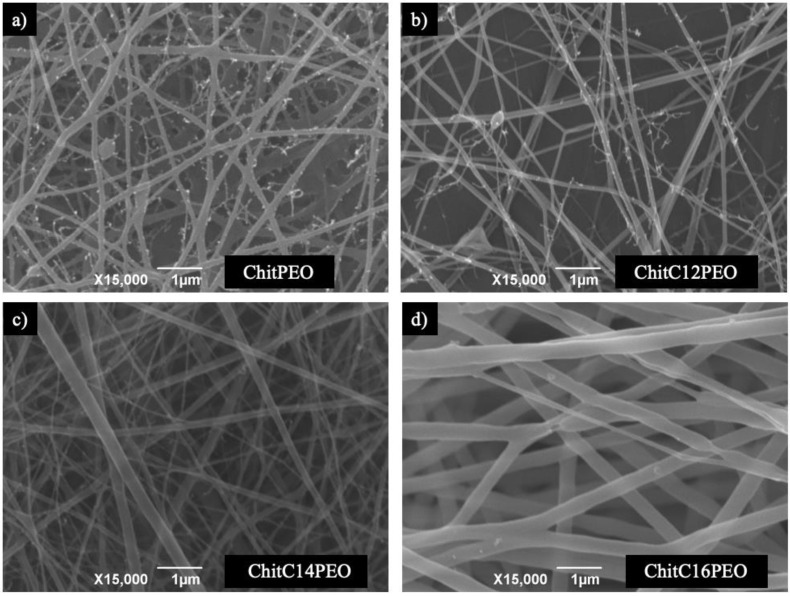
SEM images of: (**a**) matrix of ChitPEO; (**b**) matrix of ChitC12PEO; (**c**) matrix of ChitC14PEO over a layer of PEO; (**d**) matrix of ChitC16PEO over a layer of PEO.

In order to observe the morphological features of the investigated electrospun matrixes, SEM images were created (Figure 2).

In Table S1, the mean diameter values of the electrospun fibers and mean mesh dimensions are reported for each electrospun matrix. All samples show smooth fibers, with few defects, and with a dimensional variability that is not entirely negligible. In the ChitC16PEO scaffold, fiber diameter is greater with respect to the other matrices; this can be ascribed to both the lower tip-collector distance and the higher flow rate, which can contribute to arrest the jet prematurely with a larger diameter.

### 2.2. Young Modulus and Contact Angle Determinations

The literature data report that cell response depends on the elastic or viscoelastic resistance of a substrate, i.e., a cell depends on the mechanical properties of the substrates, within a few nanometers, to adhere and proliferate on a given surface. Therefore, accurate and highly local (at the nanometer scale) measurements of mechanical surface properties are needed to understand cell responses that might or might not mimic a particular tissue’s elasticity [18].

Accordingly, in order to investigate the mechanical local properties of the fibrous electrospun scaffolds synthetized in the present work, force spectroscopy, a specific application mode of atomic force microscopy (AFM), was used. AFM, indeed, is a powerful and very useful technique, involving, since its introduction, different scanning modes and modules improving its capabilities, taking profit of the different interactions between the tip and the sample surface.

In the present paper, AFM was used to map the qualitative differences in local surface properties, such as friction, adhesion and elastic modulus, measuring the force–distance curves that correlate tip-sample interaction forces and the separation distance between them. In particular, the cantilever deflection, as the tip moves in the z direction, is recorded. The response of the sample to an indentation force were employed to calculate in particular the Young modulus of peptide electrospun scaffolds, i.e., their surface stiffness. According to the mentioned literature, this information, obtained for the different tail lengths, is in turn relevant to understand cell behavior on the electrospun scaffolds.

The value distribution of Young modulus, Es, for all the investigated samples is shown in Figure S1. For pristine chitosan samples, Es values show a broad but regular Gaussian-like distribution (Figure S1a). In the case of the pristine PEO polymer, a very low Young modulus was found, of about two orders of magnitude lower (i.e., about 0.3 GPa) than those of chitosan films, with a very narrow value distribution (Figure S1b). Furthermore, it was observed that the addition of 10% of PEO to chitosan (Figure S1c) results in a considerable decrease in the Es value, from about 43.6 ± 2.2 GPa for chitosan, to an average value of 18.0 ± 0.9 GPa.

Another remarkable change of the Young modulus was found when short chains, respectively consisting in 12, 14 or 16-methylene unit tails, are attached to chitosan molecules, both for the pristine chitosan samples as well as for the chitosan-PEO samples.

**Figure 3:**
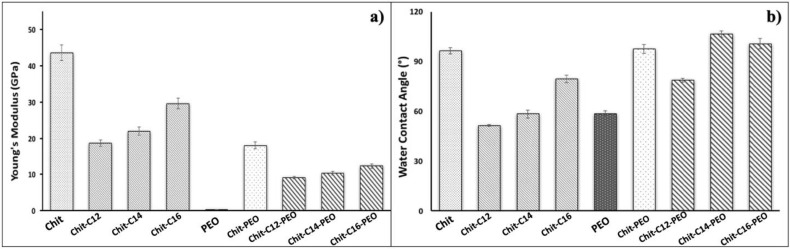
Young modulus values (**a**) and water contact angle values for the analyzed samples (**b**).

Figure S1d–f reports the Es values for chitosan-C12 (ChitC12), -C14 (ChitC14) and –C16 (ChitC16) moieties, and Figure S1g–i illustrate the Es values for ChitC12PEO, ChitC14PEO and ChitC16/PEO moieties, respectively. The results suggest that the Young modulus of thin films deposited on surface increase as the length of the aldehyde chain linked to chitosan increases (Figure 3a). In particular, the stiffness of the film increases from about 18.6 ± 0.9, 22.0 ± 1.1 and 29.6 ± 1.5 GPa, respectively, for ChitC12, ChitC14 and ChitC16, while for the mixes of ChitC12, ChitC14, and ChitC16 with PEO the Es value distribution shows a lower increase with the tail length, i.e., 9.1 ± 0.4, 10.3 ± 0.5 and 12.4 ± 0.6 GPa, respectively, for ChitC12PEO, ChitC14PEO and ChitC16PEO.

It is evident that the functionalization of the chitosan and chitosan/PEO films with the tails of different length always induces a decrease in the Young modulus, even though longer tails result in a higher modulus than shorter ones. The observed effects suggest that the presence of 10% PEO itself induces a large degradation of the chitosan macromolecular assembly cohesion and, moreover, the functionalization of the macromolecules with short chain tails seems to operate in a very similar way.

This behavior can be compared to the wettability of the investigated samples (Figure 3b). In fact, the elastic response follows the wettability trend for ChitC12, ChitC14 and Chit C16, while for ChitC12PEO, ChitC14PEO and ChitC16PEO the wettability seems independent of the tail functionalization. More in detail, the functionalization of chitosan with C12, C14 and C16 chains leads to more hydrophilic films than that of pristine chitosan, although the hydrophobicity slightly increases with the length of the hydrophobic tails. Similarly, chitosan stiffness decreases when functionalized, but *E_s_* value increases with the length of the hydrophobic tails probably due to the hydrophobic interaction among the aliphatic residues, giving a higher rigidity to the films.

On the other hand, as far as the addition of 10% PEO does not cause any significant modification of contact angle values, we argue that PEO does not segregate at the film surface: it is hidden near the surface by a layer of chitosan, according to the softening recorded by the experimental curves.

### 2.3. XPS Analysis

ChitPEO (90:10), ChitC12PEO (90:10), ChitC14PEO (70:30) and ChitC16PEO (70:30) electrospun scaffolds were analyzed by XPS spectroscopy; experimental spectra are, evidently, quite similar to each other and, for all the investigated samples, the same component peaks are detected. The measured binding energies (BEs) of the component peaks and relative attribution on the basis of the literature data [19,20,21] are reported in Table S2.

**Figure 4:**
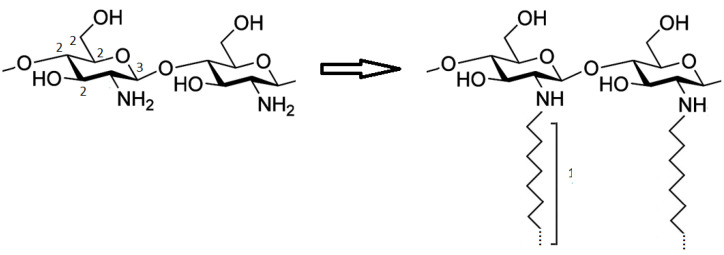
Chitosan chemical structure before and after the functionalization and assignment of the main component peaks in the C1s spectra.

The C1s spectra were fitted using three component peaks labelled C_1_ (BE = 285.0 eV), C_2_ (BE = 286.7 eV) and C_3_ (BE = 288.5 eV) in increasing BE order. The chemical structure of chitosan before and after functionalization is shown in Figure 4 and the assignment of the main component peaks is also shown.

According to the literature data [21], the first component peak C_1_ corresponds to aliphatic C–C carbons that are located in the alkyl side chains of functionalized chitosans; however, contributions to this peak result also from surface contamination that cannot be completely avoided. The peak C_2_ can be attributed to the C–O carbons of chitosan and PEO that cannot be resolved in the reported experimental conditions. According to the literature data, the contribution from the C–N amino carbon is expected at about 286.0–286.2 eV; due to its low intensity and its position between the C_1_ and C_2_ component, we are not able to resolve it from component C_2_. The third component C_3_ can be attributed to the O–C–O carbon labeled 3 in Figure 4 amide carbons, possibly resulting from only partial hydrolysis of chitin, are also expected to occur approximately at the same BE [21]. The O1s experimental spectrum results from a single peak is evidenced, having a BE in agreement with the value expected for organic O–C oxygens (BE = 532.8 eV) [19,20,21]. The N1s signal appears symmetrical and therefore was fitted with a single peak (N_1_) located at a BE of about 399.8 eV, a value typical of amine/amide/imine nitrogens; only for sample ChitPEO a second signal was detected, located at 401.8 eV, a BE value typical of protonated ammonium groups [19,20,21].

The quantitative analysis of the investigated samples was also performed; Table 1 (lines 2–5) shows the measured atomic ratios between the different element (C/O, N/O) and between atoms of the same element in different chemical environments (C_1_/C_2_, C_1_/C_3_, N_2_/N_1_).

**Figure 5:**
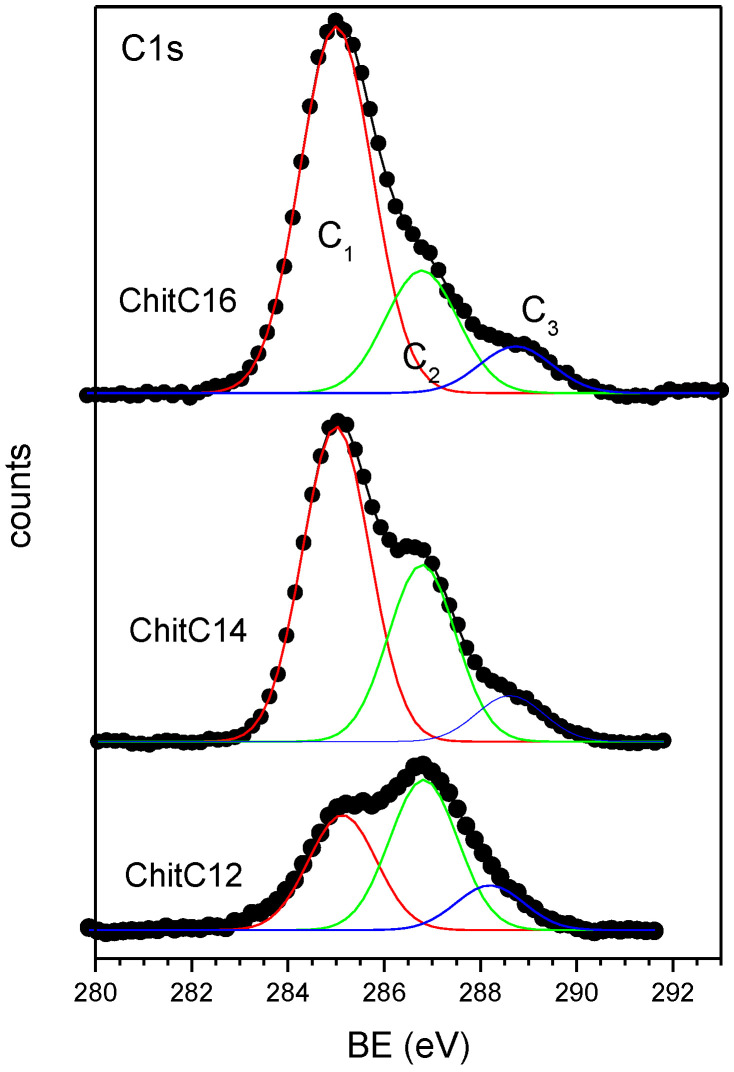
Evolution of the C1s spectra and related curve fittings for the electrospun scaffolds as a function of the aliphatic chain length. Markers represent experimental points, lines fitting components and calculated spectra.

The C1s spectrum of ChitPEO (90:10) should be free from C–C signals (C_1_); the related peak is therefore due only to surface contamination. As expected, the nitrogen main signal (N_1_) is located at a BE typical of amine nitrogens; there is also a small signal related to high BE due to the amine (N_2_) protonation; the fraction of protonated amine groups is rather low (N_2_/N_1_ ratio). The BE of O1s signal is typical of organic oxygen (O–C). In the samples containing the chitosan treated with aldehydes, the peak due to the protonated nitrogens disappears. Moreover, there is an increase in the carbon content with respect to the other elements, as evidenced by the C/O and C/N atomic ratios. By increasing the length of the aliphatic chain anchored to the nitrogen, the C/O and C/N ratios increase considerably. This effect is mainly due to the aliphatic carbon signal (C_1_) contribution: upon increasing the aliphatic chain length, the related signal (C_1_) grows in intensity compared to the C–O (C_2_) and to the O–C–O carbon (C_3_) signals, as evidenced by the increase in the C_1_/C_2_ and C_1_/C_3_ atomic ratios. The evolution of the C1s signal as a function of the aliphatic chain length is shown in Figure 5. The increase in the intensity of the C_1_ signal upon increasing the aliphatic chain length is evident; the effect is much higher than expected, taking into account the small increase in the aliphatic chain length from C12 to C16.

The XPS characterization showed, by the increase in C/N and C_1_/C_2_ ratios, that the functionalization of the chitosan with the alkyl chains took place in all proposed scaffolds.

In order to understand the reason for the unexpectedly high increase in the aliphatic carbon signal intensity and therefore in the C_1_/C_2_ atomic ratios, upon increasing the side chain length, we analyzed the pristine functionalized chitosans, before electrospinning with the PEO solution.

For all the analyzed samples, experimental spectra were fitted with the same three component peaks (C_1_–C_3_), whose assignment is the same as the one discussed for the electrospun samples. The measured atomic ratios are shown in Table 1 (lines 6–9).

The C1s signal should be absent from the chitosan spectrum; therefore, the signal detected is only due to surface contamination, which is particularly high for powder samples. The functionalization by reaction with the corresponding aldehydes produces an increase in the C/O, C/N C_1_/C_2_ and C_1_/C_3_ atomic ratios, as it was already evidenced for the electrospun matrices; all the mentioned atomic ratios increased upon increasing the alkyl chain length. However, the increase in the C/O and C/N ratios is much higher for the electrospun samples than for the pristine chitosans.

In order to account for the unexpectedly high increase in the aliphatic C_1_ component and in the C/O, C/N atomic ratios for the electrospun samples, we can suppose that in these samples hydrophobic alkyl chains tend to be oriented towards the fibers surface, thus enhancing the C_1_ peaks spectral contribution for a surface-sensitive technique, such as XPS.

### 2.4. Dynamic Mechanical Analysis

The dynamic mechanical analysis showed that the values of the storage (G′) and loss (G″) moduli increased with frequency for all chitosan solutions (Chit, ChitC12, ChitC14, and ChitC16).

**Figure 6:**
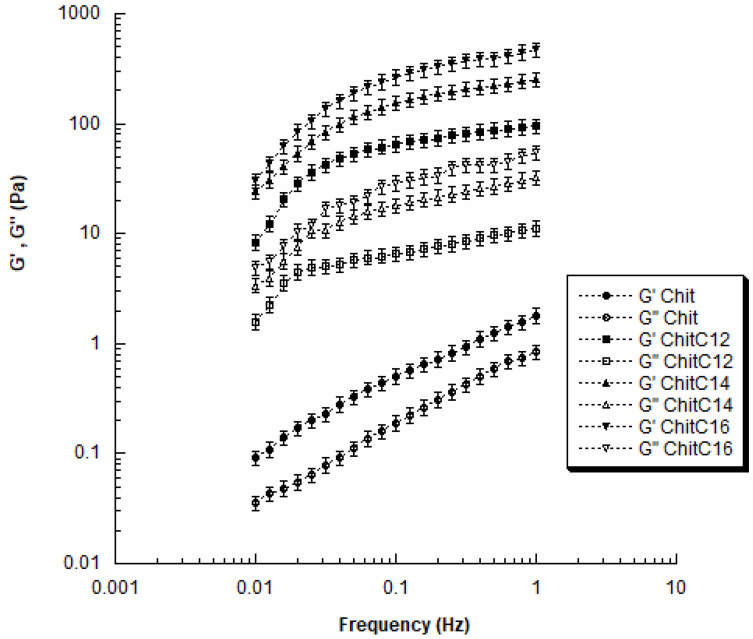
Dynamic mechanical analysis. Results from small amplitude oscillatory shear tests: storage modulus (G′) and loss modulus (G″) as function of frequency for Chit, ChitC12, ChitC14, and ChitC16. Data are reported as mean value, and error bars represent the standard deviation. The results were analyzed using ANOVA followed by Bonferroni post hoc tests. The level of significance was set at *p* < 0.05.

Furthermore, G′ values were always higher than the G″ ones in the investigated frequency range (Figure 6).

In general, an increase in both dynamic moduli was observed with the increase in chain length (Figure 6). In particular, as the frequency increased from 0.01 to 1 Hz; Chit showed G′ values ranging from 0.09 ± 0.01 Pa to 1.8 ± 0.2 Pa, which were significantly lower than those found for ChitC12 (from 8.4 ± 0.9 Pa to 96.3 ± 9.8 Pa).

Moreover, by further increasing the chain length, G′ increased as values ranging from 24.0 ± 2.2 Pa to 251.0 ± 20.5 Pa and from 32.7 ± 2.6 Pa to 466.0 ± 35.7 Pa were achieved for ChitC14 and ChitC16, respectively. The observed differences were statistically significant.

Clearly, the long chain has a strong effect on the behavior of polymer solutions, also leading to the changes in morphological and structural features.

In a polymer solution, the polymer chains are more or less isolated according to the solution concentration. The conformation of the molecules and the hydrodynamic volume strongly affect the physical, flow and dynamic mechanical properties.

The obtained findings should be also explained in terms of chain entanglements and interactions. It is well known that, at a fixed polymer concentration, an increase in chain length leads to a restriction in terms of freedom of movement for the individual chains, as consequence of an increased number of entanglements and interactions. Thus, a decrease in chain length led to a reduction in the storage modulus (Figure 6) as a result of an increased flexibility of the chain due to a reduction in the number of interactions and entanglements between the molecules.

### 2.5. Bioassays

#### 2.5.1. Bacteriostatic/Bactericidal Properties of Electrospun Functionalized Chitosan Scaffolds

*E. Coli* and *S. Aureus* bacteria did not grow in contact with all the electrospun scaffolds, revealing neither bacteriostatic nor bactericidal properties. The results are reported in Figure S2 of the Supplementary Material.

#### 2.5.2. Cellular Properties of Electrospun Functionalized Chitosan Scaffolds

**Figure 7:**
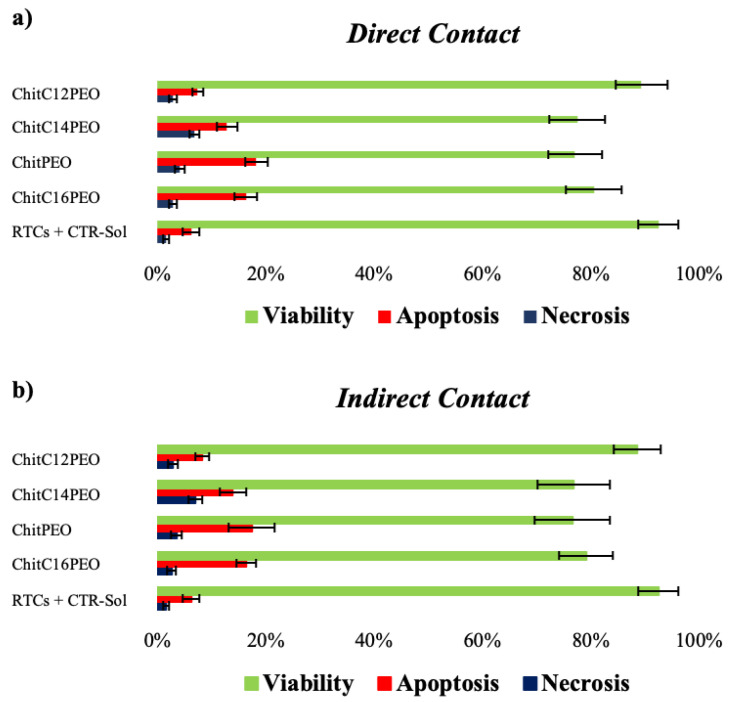
Percentage of Direct Contact (**a**) and Indirect Contact (**b**) viability, apoptosis and necrosis between RTCs in CTR-Sol incubated 24 h with the 4 test devices and RTCs incubated only with CTR-Sol.

In Direct Contact tests, renal tubular cells (RTCs) showed a good growth, as onto CTR-Sol (complete RTCs medium, paragraph 4.2.10.1). The use of RTCs is justified by our previous studies [22]; they have shown a positive efficiency in the release of cytokines when subjected to external stressors. The quantitative analysis using Annexin V/PI, cytofluorometric assay, showed a difference in terms of viability, apoptosis and necrosis between RTCs incubated with the four different scaffolds and RTCs incubated only with CTR-Sol (Figure 7a). ChitPEO, ChitC14PEO and ChitC16PEO resulted in higher mortality than CTR-Sol, while ChitC12PEO mortality was similar to CTR-Sol. These differences were found both in Direct Contact and Indirect Contact tests (Figure 7a,b).

**Figure 8:**
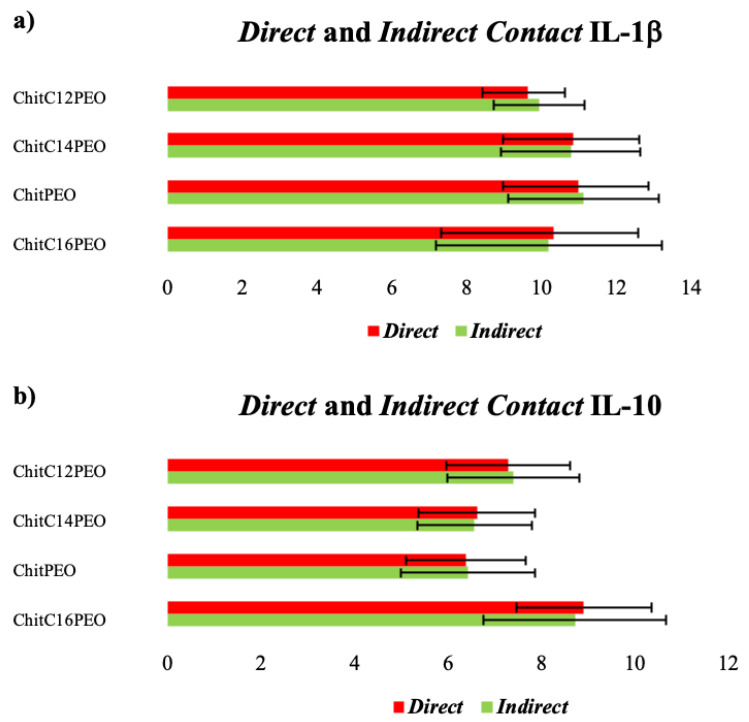
Direct and Indirect Contact IL-1β (**a**) and IL-10 (**b**) levels. Results are expressed in pg/mL.

Pro- and anti-inflammatory cytokine levels (IL-1β and IL-10) showed no differences both in Direct Contact and Indirect Contact tests (Figure 8). We analyzed pro-inflammatory Il-1β and anti-inflammatory IL-10 cytokines to see if the materials caused an effect on RTCs. As there were no pro- or anti-inflammatory effects, we did not consider proceeding with further investigations.

Non-elevated values seem to indicate that the four scaffolds did not have any inflammatory and/or anti-inflammatory effects on RTCs.

**Figure 9:**
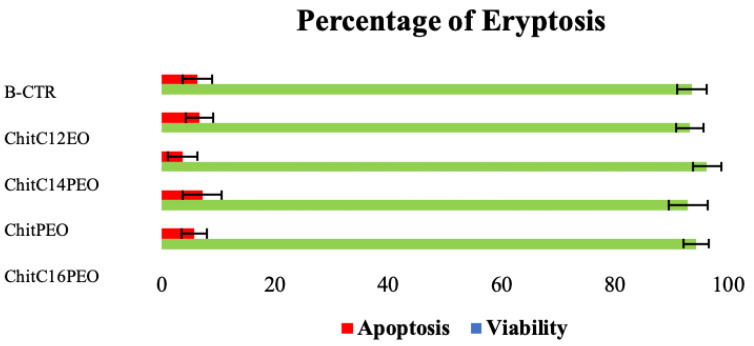
Percentage of eryptosis after 24 h of incubation with 4 electrospun scaffolds and without (B-CTR).

As shown in Figure 9, after 24 h incubation, flow cytometry analysis showed no differences in eryptosis among the four scaffolds and the blood control (B-CTR). Pro- and anti-inflammatory cytokine levels (IL-1β and IL-10) showed no substantial differences among the four different scaffolds and in comparison, with B-CTR (Figure S3).

Unlike the other factors, myeloperoxidase (MPO) levels were higher in ChitC14PEO and ChitC16PEO (respectively, 1205.0 and 925.4 pg/mL), lower than the first two for ChitPEO and ChitC12PEO (respectively, 381.5 and 306.8 pg/mL), and normal for B-CTR (87.7 pg/mL) (Figure S4).

It is well known that chitosan, in direct contact with blood, promotes plasma protein adsorption, platelet adhesion and thrombolysis [23]. Serum in contact with chitosan-PEO (80:20) membrane did not cause sheep erythrocytes lysis [24].

All functionalized chitosan matrices showed no cytotoxicity induced in blood cells, no cell adhesion enhancement, no antimicrobial activity and no inflammation response.

## 3. Conclusions

In this study, a method for the preparation of fibrous scaffolds composed of chitosan functionalized with dodecanal or tetradecanal or hexadecanal was carried out.

The XPS analyses demonstrated the efficacy of the chitosan functionalization. To obtain virtually defect-free fibrous scaffolds, a blend of functionalized chitosan and PEO was used. AFM analyses performed on films of pristine or PEO-added materials demonstrated that both the addition of PEO and the functionalization with hydrophobic tails dramatically change the properties of chitosan. In particular, the addition of PEO lowered the Young modulus of the chitosan film. A similar result was also observed as a response to functionalization, proving that both PEO and the hydrophobic tails (in particular C12) additions produced a loss of macromolecular cohesion. Surprisingly, the evaluation of the contact angle indicated that the addition of PEO did not substantially modify the wettability of chitosan whilst the functionalization led to more hydrophilic films. The DMA analysis allowed the study of the significant effect of the chain length on the viscoelastic properties of chitosan solutions in the investigated frequency range (e.g., G′ ranging from 0.09 ± 0.01 Pa to 1.8 ± 0.2 Pa–Chit, from 8.4 ± 0.9 Pa to 96.3 ± 9.8 Pa–ChitC12, from 24.0 ± 2.2 Pa to 251.0 ± 20.5 Pa–ChitC14, and from 32.7 ± 2.6 Pa to 466.0 ± 35.7 Pa–ChitC16).

The biological assays aimed at studying the interaction of the electrospun membranes with bacteria (both Gram + and Gram−), cells of the renal tubule and blood. The results agreed in indicating all matrices as constitutionally sterile, but neither bactericidal nor bacteriostatic. Moreover, such matrices did not show adhesive property with respect to renal tubular cells. No erythrocytosis phenomena or immunological effects were observed. All these findings combine to define these scaffolds as promising materials for biomedical devices, such as hemodialysis filters, hemopurifiers and blood vessel substitutes for regenerative medicine. The current study represents a potential opening for the application of electrospun chitosan for these new medical devices.

## 4. Materials and Methods

### 4.1. Materials

Acetic acid and poly(ethylene oxide) (average MV 900,000 Da) were supplied by Sigma-Aldrich (Steinheim, Germany). Chitosan 70/1000 (deacetylation grade between 67.7 and 72.5%; viscosity = 1000 mPas) was obtained from Heppe Medical Chitosan GmbH (Saale, Germany). Ethanol and sodium cyanoborohydride were purchased from Fluka (Steinhem, Germany). Dodecanal, tetradecanal and hexadecanal were obtained from Toronto Research Chemicals (Toronto, Canada). Sodium phosphate was supplied by Labscan Carlo Erba (Milan, Italy) and sodium chloride by Prolabo (Briare, France).

### 4.2. Methods

#### 4.2.1. Preparation of ChitC12, ChitC14 and ChitC16

Three different modified types of chitosan were prepared by reaction between chitosan and three aldehydes containing different aliphatic tails (dodecanal, tetradecanal and hexadecanal) as follows: 4 g of chitosan were slowly added in a 0.2 M acetic acid solution under magnetic stirring. The solution was diluted in ethanol (150 mL) and the pH was adjusted at 5.1 with 1 M sodium hydroxide solution in order to prevent chitosan precipitation. Each aldehyde (0.257 g) was dissolved in ethanol (10 mL, 0.025 g/mL) and then added to the chitosan solution. An excess amount of sodium cyanoborohydride (4.3 g) was added at room temperature under stirring for 24 h. The reacted chitosan was precipitated from solution by increasing the pH up to 7 using NaOH solution (1 M) and adding an excess of ethanol. The precipitate was filtered in a gooch and washed with ethanol to remove the unreacted aldehyde and sodium cyanoborohydride. The resulting product (ChitC12 or ChitC14 or ChitC16) was dried under vacuum at room temperature.

#### 4.2.2. Preparation of Polymer Solutions for Electrospinning

As reported in [25], the electrospinning solution of pristine chitosan and PEO was prepared at 1.6% *w/w* polymer/solution concentration, where the polymer weight was composed by 10% PEO and 90% chitosan; the solvent used was 90% *v*/*v* acetic acid solution in water. This method was suitable for preparing ChitC12 solution but not for ChitC14 and ChitC16: for the latter, the obtained solutions were not properly electrospinnable. Therefore, a salt was added for increasing solution conductivity. A total of 0.8773 g of sodium chloride and 0.138 g of sodium phosphate were dissolved in 100 mL of distilled water and pH was adjusted to 7.4. The polymeric solutions for the electrospinning of ChitC14 or ChitC16 were obtained according to the following procedure: 0.07 g of PEO were dissolved in 4.7 mL of acetic acid solution (90:10) and separately 0.07 g of ChitC14 (or ChitC16) were added to 4.7 mL of acetic acid solution (90:10). Then, 2 mL of PEO solution were added to ChitC14 (or ChitC16) solution to obtain a final solution composed by 70% of functionalized chitosan and 30% of PEO. Eventually, the solution was diluted with 1 mL of acetic acid/buffer solution (90:10).

#### 4.2.3. Electrospinning Process

The electrospinning conditions used to obtain scaffolds of nanofibers are reported in Table S3.

Since the layers of electrospun ChitC14PEO and ChitC16PEO were excessively thin and difficult to be detached from the collector, they were deposited on a first layer of electrospun PEO (5% PEO *w*/*w* in water).

#### 4.2.4. Scanning Electron Microscopy

Electrospun scaffolds were sputter coated with gold (EMITECHK950x Turbo Evaporator, EBSciences, East Granby, CT, USA) and observed under SEM (Cambridge Stereoscan 440 SEM, Cambridge, UK). Images were recorded at 15,000× magnifications with an accelerating voltage of 20 kV. The diameter range of the fabricated nanofibers was manually measured using a commercial imaging software (ImageJ, National Institutes of Health, Bethesda, MD, USA). For each sample, three representative images of three different areas were chosen.

#### 4.2.5. Preparation of Polymer Solutions for Contact Angle Measurements, Young Moduli Determination and DMA Analysis

All solutions for films preparation were prepared with 1.6% *w*/*w* polymer/solution concentration, where polymer weight was composed by 100% PEO (PEO); 100% Chitosan (Chit); 100% ChitC12 (ChitC12); 100% ChitC14 (ChitC14); or 100% ChitC16 (ChitC16). Other 1.6% w/w solutions were prepared considering the polymer weight composed by 10% of PEO and 90% of chitosan (ChitPEO); 90% ChitC12 (ChitC12PEO); 90% ChitC14 (ChitC14PEO); or 90% ChitC16 (ChitC16PEO). The solvent used was 90% *v*/*v* acetic acid solution. For contact angle measurements and Young moduli determination, the solutions were deposited on glass surfaces and left to dry at 25 °C, whilst for DMA analysis the experiments were carried out directly on the different polymer solutions.

#### 4.2.6. Water Contact Angle Measurements

Surface wettability was measured by static water contact angle. The OCA30 instrument (Dataphysics, Filderstadt, Germany) was used at 25 °C and under 65% relative humidity. The measurements were carried out on each polymer film deposited on a glass surface. This analysis was not carried out on electrospun matrices in order to avoid the effect due to the porous fibrous structure that could lead to a difficult interpretation or misleading. Then, 2 μL ultrapure water drops were applied on different zones of each sample surface and the static contact angle was measured on both sides of the two-dimensional projection of the droplet by digital image analysis. At least three measurements were made for each sample, and values were averaged.

#### 4.2.7. AFM Analysis

AFM analyses were carried out on different polymer films in order to evaluate separately the effects of hydrophobic tails functionalization and PEO introduction. An analogue study on electrospun matrices was not feasible because ChitC12, ChitC14 and ChitC16 could not be electrospun without PEO. The Young modulus was calculated from the collected force–distance curves measured with the NTEGRA AFM (NT-MDT, Moscow, Russia). Stiff single crystal silicon cantilevers with a symmetric tip shape were used (model Tap300Al-G, BudgetSensors, Bulgaria: nom. Frequency 300 kHz, nom. Spring constant 40 Nm^−1^, tip radius < 10 nm). AFM measurements were in triplicate. The probe was characterized by measuring the cantilever spring constant by the Sader method [26]. Each probe was calibrated by performing a force-curve on a hard-cleaned substrate (<100> silicon wafer), where no indentation occurred. Probe calibration was needed to calculate their sensitivity and the spring constant. The Young modulus was obtained from the experimental force–distance curves by the Derjaguin–Müller–Toporov (DMT) model [27], using the following equation:

(1) F+Fad=4Es3(1−νs2)R12δ32,

where *F* is the applied force; *F_ad_* is the adhesion force; *Es* is the Young modulus; *ν*_s_ is the Poisson’s ratio for the sample; *R* is the radius of the spherical indenter; and *δ* is the elastic indentation depth. Each surface was indented in different areas and about three hundred curves were acquired. The tip-sample approaching rate was set to 0.3 µms^−1^ for all force curves. The Young modulus was calculated by fitting experimental curves with the DMT model in the elastic region. Elastic modulus distribution was assessed with OriginPro 8.5 software.

#### 4.2.8. XPS Measurements

XPS studies were performed on electrospun matrices using an in house designed instrument consisting of one preparation and one analytical chamber separated by a gate valve. The analytical chamber is equipped with a six degrees of freedom manipulator and a 150 mm mean radius hemispherical electron analyzer with five-lens output system combined with a 16-channel detector. Measurements were performed at normal take-off (θ = 90°). Samples were introduced in the preparation chamber and outgassed overnight under vacuum (about 10^−8^ Torr), before being transferred into the analytical chamber. Typical vacuum pressure into the analytical chamber was in the range 10^−9^–10^−10^ Torr.

Mg Kα non-monochromatized X-radiation (hν = 1253.6 eV) was used for recording C1s, O1s and N1s core level spectra, with a pass energy of 25 eV. Measurements were performed on at least two different specimens for each sample in order to check data reproducibility; mean values are reported. Analysis was carried both on the electrospun matrices and on the pristine chitosan chosen as reference; these latter were analyzed as powders deposited on an adhesive tape on Ti substrates.

The measured binding energies (±0.1 eV) were calibrated to the C1s signal of aliphatic C–C carbons located at a binding energy BE = 285.0 eV [19]. Experimental spectra were analyzed by curve fitting using Gaussian curves. Atomic ratios (±10%) were calculated from peaks areas using Scofield’s cross section as sensitivity factors.

#### 4.2.9. DMA Analysis

Dynamic mechanical analysis was performed on different chitosan solutions (Chit, ChitC12, ChitC14, and ChitC16) in order to evaluate separately the effects of hydrophobic tails functionalization and PEO introduction. An analogue study on electrospun matrices was not feasible because ChitC12, ChitC14 and ChitC16 could not be electrospun without PEO.

In particular, dynamic oscillatory shear measurements were carried out at 37 °C using a rheometer (Gemini, Bohlin Instruments, Malvern Panalytical Ltd, Malvern, UK) with a parallel plate. Serrated parallel plates with a diameter of 15 mm were employed to avoid slippage.

First, the linear viscoelastic region was determined, and small amplitude oscillatory shear tests were successively performed in the frequency range from 0.01 to 1 Hz.

The storage or elastic modulus (*G′*) and the loss or viscous modulus (*G″*) were evaluated as follows:

(2) $$G^{\prime }=\frac{\tau_{0}}{\gamma_{0}}\cos\delta ,$$

(3) $$G\prime \prime =\frac{\tau_{0}}{\gamma_{0}}\sin\delta$$

where *δ* represents the phase shift between the input and the output signals, whilst *γ*_0_ and *τ*_0_ and are the strain and stress amplitudes, respectively.

The data were reported as mean value ± standard deviation and analyzed using ANOVA followed by Bonferroni post hoc tests. The level of significance was set at *p* < 0.05.

#### 4.2.10. Biological Assays

##### RTCs Culture

Primary cultures of human proximal renal tubular epithelial cells were obtained from kidneys explanted from patients affected by renal carcinomas.

An immortalized human proximal renal tubular cells (RTCs) line was generated by infection with a hybrid Adeno5/SV40 virus. The purity of primary cultures was assessed according to published criteria [28]. RTCs were grown in complete liquid phase medium (RPMI 1640 with stable L-glutamine) supplemented with 10% heat-inactivated (30 min at 56 °C) fetal bovine serum, 100 IU/mL penicillin, and 100 μg/mL streptomycin (Sigma-Aldrich, Merck KGaA, Saint Louis, USA) (CTR-Sol). RTCs were maintained in an incubator under controlled atmosphere and temperature (5% CO_2_ at 37 °C) and passaged at 80% confluence checked by inverted microscope.

##### Test by Direct and Indirect Contact

In the Indirect Contact tests, 4 specimens of electrospun scaffolds (ChitPEO (90:10), ChitC12PEO (90:10), ChitC14PEO (70:30) electrospun on a PEO layer, and ChitC16PEO (70:30) electrospun on a PEO layer) with the same 0.4 × 0.4 cm^2^ surface area were incubated at 37 °C and 5% CO_2_ for 24 h in 2 mL of CTR-Sol and in 2 mL of RPMI solution plus 1 × 10^4^ RTCs (Direct Contact). Thereafter, part of CTR-Sol (0.5 mL) incubated with electrospun scaffolds was withdrawn and incubated for other 24 h with RTCs.

In the Direct Contact test, the solutions with RTCs were centrifuged for 7 min at 3500 rpm, and supernatants were immediately separated from RTCs, then frozen and used in a second phase to assess the inflammatory factor IL-1β and anti-inflammatory factor IL-10.

RTCs viability, apoptosis and necrosis were assessed using Annexin V-Fluorescein isothiocyanate (FITC) kit (Beckman Coulter, Brea, CA, USA) according to the manufacture’s protocol. This kit is based on the binding properties of Annexin V to phosphatidylserine and on DNA-intercalating capabilities of propidium iodide (PI). The analysis was performed using a Navios flow cytometer (Beckman Coulter, Brea, CA, USA). The biparametric analysis revealed three distinct populations: viable cells with low phenyl-isothiocyanate (FITC) and low PI signals, apoptotic cells with high FITC and low PI signals, and necrotic cells with high FITC and high PI signals. A minimum of 15,000 events were collected for each sample.

After further 24 h, solutions from indirect contact test were centrifuged for 7 min at 3500 rpm, and supernatants were immediately separated from RTCs, then frozen and used in a second phase to assess the inflammatory factor IL-1β and anti-inflammatory factor IL-10.

In the second phase, the quantitative determination of IL-1β and IL-10 in direct and indirect contact test solutions was performed by the Human Instant enzyme-linked immunosorbent assay (ELISA) kit (eBioscience, San Diego, CA, USA). Cytokine determinations were performed according to the manufacturer’s protocol and instructions. Optical density was read by VICTOR X4 Multilabel Plate Reader (PerkinElmer Life Sciences, Waltham, USA) at 450 nm. The concentration of cytokines was calculated from the standard curve according to the manufacturer’s protocol. IL-1β, IL-10 and myeloperoxidase (MPO, marker for oxidative stress) concentrations were calculated from the standard curve according to the manufacture’s protocol. All tests were performed in triplicate.

##### Test on Extracts with Blood

Five peripheral venous blood samples were collected from a healthy volunteer; four samples were used for test devices and one sample as control (B-CTR). Blood samples were collected in EDTA tubes and subsequently each material, with the same surface (0.8 × 0.4 cm^2^), was placed within a tube of blood sample. EDTA tubes with test devices were gently mixed at 37 °C for 24 h.

For assays with red blood cells (RBC), blood was centrifuged for 7 min at 3500 rpm and the plasma, buffy coat, and uppermost erythrocytes were removed and discarded. The remaining erythrocytes were washed with PBS at room temperature and immediately stained with Annexin-V kit (Beckman Coulter, Brea, CA, USA), then evaluated by flow cytometry. The analysis was performed using a Navios flow cytometer (Beckman Coulter, Brea, CA, USA).

The plasma was frozen and used in a second phase to check the inflammatory factor IL-1β and anti-inflammatory factor IL-10 and MPO. All tests were performed in triplicate.

##### Sterilization Test with Ethanol 20%

In order to evaluate the sterilization ability of the ethanol 20% solution, two strains were used: E. coli and S. aureus. Each strain was gradually diluted (2 McF, 1 McF, 0.5 McF, 0.25 McF) and 1 mL from each diluted solution was centrifuged at 14,000 rpm for 5 min. The supernatant was removed. The remaining pellet was treated with 1 mL of ethanol 20% (made in hospital pharmacy, Vicenza Hospital, Vicenza, Italy). At this stage, the solutions were vortexed to resuspend bacteria. After 10 min, 20 min, 30 min, 1 h, each alcoholic/bacterial solution was plated with 1 µL calibrated loop into blood agar medium (BioMerièux, Marcy l’Etoile, France). Agar plates were incubated for 24 h at 37° to promote bacteria growth.

##### Sterilization Test with Ethanol 70%

In order to evaluate the sterilization ability of the ethanol 70% solution, two strains were used: *E. coli* and *S. aureus*. Each strain was gradually diluted (2 McF, 1 McF, 0.5 McF, 0.25 McF) and 1 mL from each diluted solution was centrifuged at 14,000 rpm for 5′. The supernatant was removed. The remaining pellet was treated with 1 mL of ethanol 70% (made in hospital pharmacy, Vicenza Hospital). At this stage, solutions were vortexed to resuspend bacteria. After 10 min, 20 min, 30 min, 1 h, each alcoholic/bacterial solution was plated with 1 µL calibrated loop into blood agar medium (BioMerièux). Agar plates were incubated for 24 h at 37 °C to promote bacteria growth.

##### Membrane Sterilization Test

In order to assess the ethanol 70% solution sterilization ability over the membranes, squared samples (5 mm × 5 mm) were cut from each membrane. Each sample was immerged for 10 min into ethanol 70% solution. After 10 min, the sample was plated in a blood agar medium (BioMerièux) and incubated for 24 h at 37 °C.

##### Antibacterial Properties

In order to evaluate membrane antibacterial properties, one circular sample (5 mm diameter) was cut from each membrane. The material was treated with ethanol 70% (made in hospital pharmacy, Vicenza Hospital) and after 10 min was plated in neutered Petri plates (BioMerièux) and dried up for 24 h.

##### 1st Antibacterial Property Test

In order to evaluate membrane antibacterial properties, 2 strains ATCC were used: *E. coli* and *S. aureus*. After being stored at −20 °C, they were thawed completely and then plated into a blood agar medium (BioMerièux); finally, they were incubated for 24 h at 37 °C. After incubation, the strains were used for getting 0.5 McF solutions. Thus, bacterial solutions were plated in a Mueller–Hinton agar medium (BioMerièux). At this stage, previously prepared membranes were plated in seeded medium to evaluate possible inhibition haloes. The plates were incubated for 24 h at 37 °C.

##### Second Antibacterial Property Test

For the second antibacterial test, 2 rectangular specimens (0.5 mm × 10 mm) were obtained. All the above-mentioned steps were repeated, but the membranes were plated for 10 min in a portion of the plate and after plated in a final locus.

##### Membrane Negative Control

A second test was repeated on adsorption paper pieces of the same dimensions to assess if smaller bacterial growth is due to inhibition contact or to the antibacterial properties of the investigated materials.

##### Membrane Sterilization Test

In order to evaluate membrane antibacterial properties after UV sterilization for 12 h, 2 strains ATCC were used: E. coli and S. aureus. After being stored at −20 °C, they were thawed completely and then plated into a blood agar medium (BioMerièux); finally, they were incubated for 24 h at 37 °C. After incubation, the strains were used to obtain 0.5 McF solutions. Then, the bacterial solutions were plated in aMueller–Hinton agar medium (BioMerièux). At this stage, the 24 h previously prepared membranes were plated in a seeded medium and the plates were incubated for 24 h at 37 °C.

##### Liquid Test for Evaluating Membrane Antibacterial Properties

For the liquid evaluation of membrane antibacterial properties, 2 strains ATCC were used: E. coli and S. aureus. After being stored at −20 °C, they were thawed completely and plated into a blood agar medium (BioMerièux); finally, they were incubated for 24 h at 37 °C. After incubation, the strains were used to obtain 1 McF solutions. A total of 500 µL from each bacterial suspension were added with 500 µL of Brain Heart Infusion medium (B.H.I., BLL™ Brain Heart Infusion, Becton Dickinson, East Rutherford, USA) to obtain a 0.5 McF solution. Therefore, 10 Eppendorfs were prepared, each containing 1 mL of this solution (5 with E.c. 0.5 McF solution and 5 with S.a 0.5 solution). Then, 8 membrane portions, 24 h previously sterilized and prepared, were placed into the bacterial solution. The plates were then incubated at 37 °C. From each Eppendorf, previously vortexed for resuspending bacteria, an aliquot was plated for 0, 1, 8, 24 h. Each aliquot was plated with 1 µL calibrated loop into a blood agar medium (BioMerièux). The agar plates were finally incubated at 37 °C.

## Figures and Tables

**Table 1:** Atomic ratios for electrospun scaffolds and reference samples.

Sample	C/O	C/N	C_1_/C_2_	C_1_/C_3_	N_2_/N_1_
ChitPEO (90:10)	1.78	14.2	0.7	2.1	0.10
ChitC12PEO (90:10)	2.44	26	0.76	2.6	-
ChitC14PEO (70:30)	3.45	117	1.8	6.8	-
ChitC16PEO (70:30)	4.03	172	3.0	7.8	-
Chit	2.49	15.8	1.63	4.54	0.15
ChitC12	2.78	19.4	2.25	5.52	-
ChitC14	2.99	20.4	3.48	6.79	-
ChitC16	3.37	29.5	5.17	7.53	-

## Data Availability

The data presented in this study are available within this article and in Supplementary Material.

## Supplementary Materials

The following are available online at www.mdpi.com/article/10.3390/gels8020113/s1. Table S1: Mean diameter of fibers and mean mesh dimension ± SD; Figure S1: Frequency distribution of Young modulus; Table S2: Measured BEs and assignment of the XPS component peaks; Figure S2: Bacteriostatic/bactericidal properties of the scaffolds; Figure S3: IL-1b levels and IL-10 levels after 24 h; Figure S4: MPO levels after 24 h; Table S3: Electrospinning conditions used to obtain scaffolds of nanofibers.

## Abbreviations

C12	hydrocarburic tails with 12 methylene units
C14	hydrocarburic tails with 14 methylene units
C16	hydrocarburic tails with 16 methylene units
DA	acetylation degree
SEM	scanning electron microscope
XPS	X-ray photoelectron spectroscopy
DNA	deoxyribonucleic acid
DMA	dynamic mechanical analysis
AFM	atomic force microscopy
RTCs	renal tubular cells
WCA	water contact angle
PEO	polyethylene oxide
ChitC12	Chitosan-C12
ChitC14	Chitosan-C14
ChitC16	Chitosan-C16
ChitC12PEO	Chitosan-C12 with the addition of PEO
ChitC14PEO	Chitosan-C14 with the addition of PEO
ChitC16PEO	Chitosan-C16 with the addition of PEO
ChitPEO	Chitosan with the addition of PEO
Es	values distribution of Young modulus
G′	storage modulus
G″	loss modulus
ANOVA	analysis of variance
CTR-Sol	complete RTCs medium
MPO	myeloperoxidase
B-CTR	blood control
DMT	Derjaguin–Müller–Toporov
FITC	Phenyl-isothiocyanate
RBC	red blood cells
