# Supplementary material for: Electrospun Chitosan Functionalized with C12, C14 or C16 Tails for Blood-Contacting Medical Devices
Source: Gels. 2022 Feb 12;8(2):113. doi: 10.3390/gels8020113 (PMC8872026; doi:10.3390/gels8020113)
Supplement: Supplementary file 1 [file gels-08-00113-s001.zip › gels-1508672-supplementary.pdf]

gels

MDPI

Supplementary Materials

Article

Electrospun Chitosan Functionalized with C12, C14 or C16 Tails for Blood-Contacting Medical Devices

Monica Dettin [1], Martina Roso [1], Grazia M. L.Messina [2], Giovanna Iucci [3], Valentina Peluso [4], Teresa Russo [4], Annj Zamuner [1,*], Marta Santi [3], Sabrina Milan Manani [5], Monica Zanella [5], Chiara Battocchio [3], Giovanni Marletta [2], Michele Modesti [1], Mario Rassu [5], Massimo De Cal [5], Claudio Ronco [5,6]

Table S1. Mean diameter of fibers and mean mesh dimension ± SD.

Electrospun scaffold	Mean diameter (nm)	Mean mesh dimension (nm)
**ChitPEO (90:10)**	130.5±33.3	808.7±311.2
**ChitC12PEO (90:10)**	127.9±27.8	768.7±380.9
**ChitC14PEO (70:30)**	172.5±65.2	643.5±226.5
**ChitC16PEO (70:30)**	331.8±71.5	1156.0±820.4

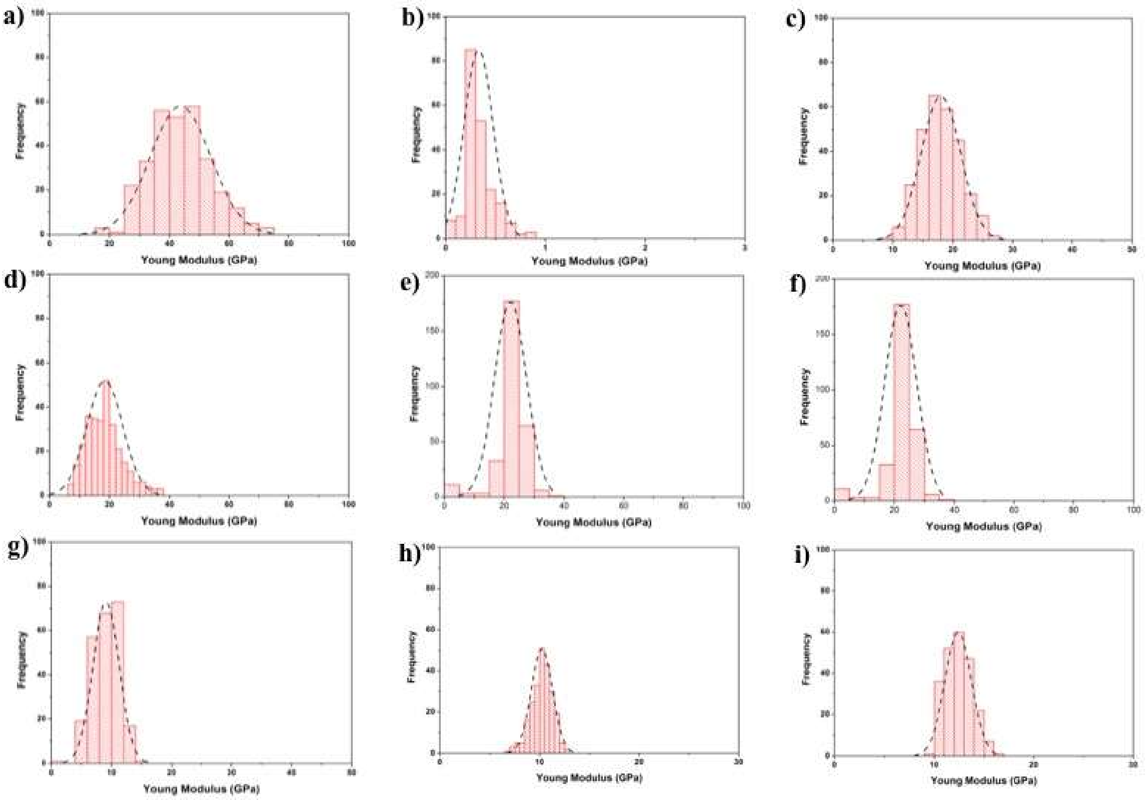

Figure S1. Frequency distribution of Young's modulus of a) Chitosan, b) PEO, c) ChitPEO, d) ChitC12, e) ChitC14, f) ChitC16, g) ChitC12PEO, h) ChitC14PEO and i) ChitC16PEO.

Table S2. Measured BEs and assignment of the XPS component peaks.

Spectrum	Components	BE (eV)	Assignment
	C_1	285.0±0.1	C-C
**C1s**	C_2	286.7±0.1	C-O, C-N
	C_3	288.5±0.1	O-C-O, N-C=O
**N1s**	N_1	399.8±0.1	unprotonated
**O1s**		532.8±0.1	O-C

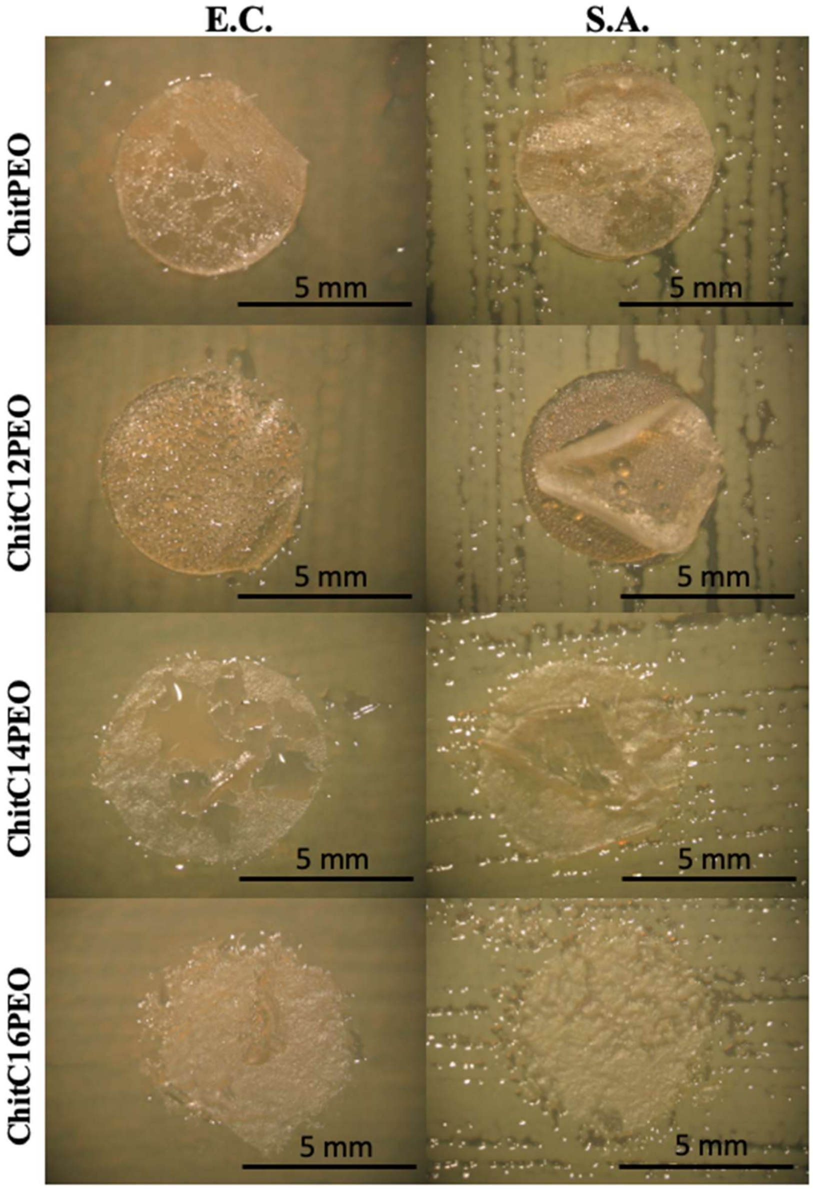

Figure S2. Bacteriostatic/bactericidal properties of the scaffolds. After removal of a circular portion of the every four different electrospun scaffolds (ChitPEO, ChitC12PEO, ChitC14PEO and ChitC16PEO), E.coli and S. aureus were plated in Mueller-Hinton agar and incubated with the four electrospun patches for 24 h at 37°. No growth in contact with all the electrospun scaffolds.

gels

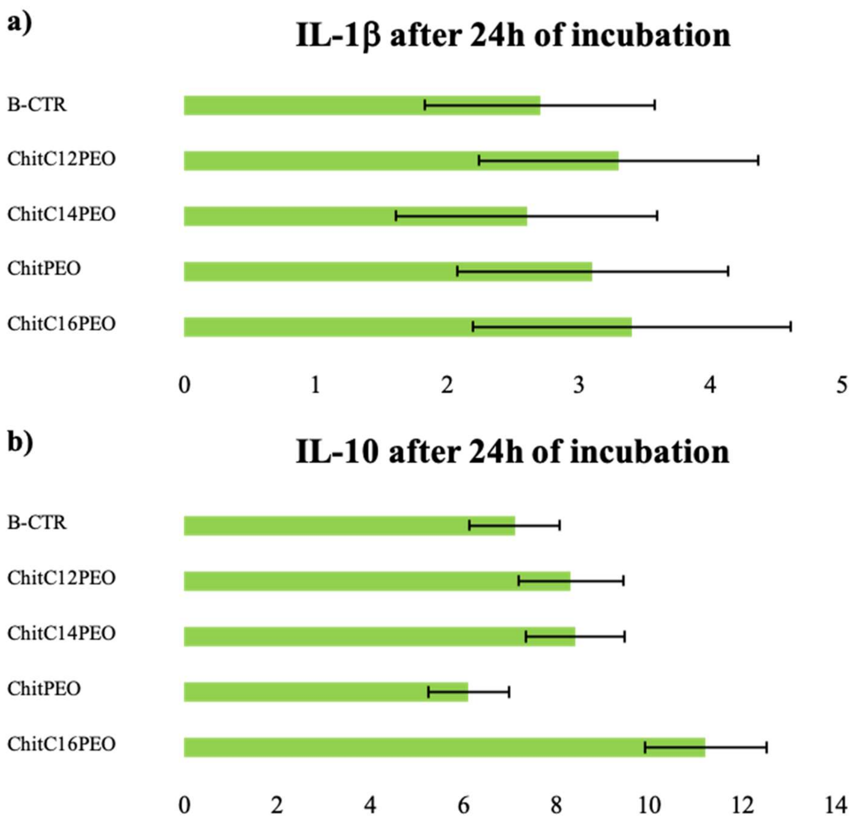

Figure S3. IL-1b levels (**a**) and IL-10 levels (**b**) after 24h of healthy volunteer blood incubation with 4 test devices and without (B-CTR). Results are expressed in pg/mL.

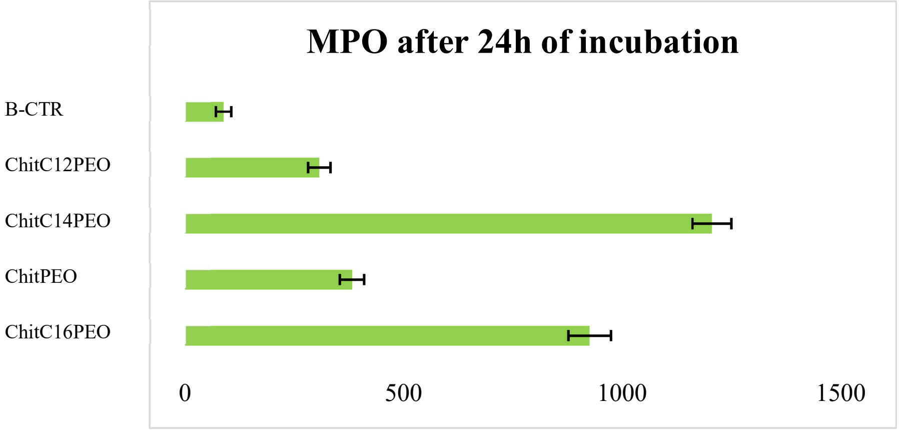

Figure S4. MPO levels after 24h of healthy volunteer blood incubation with 4 test devices and without (B-CTR). Results are expressed in pg/mL.

Table S3. Electrospinning conditions used to obtain scaffolds of nanofibers.

	Needle (G)	Total volume (ml)	Flow rate (ml/h)	Voltage (kV)	Tip to collector distance (cm)	Temperature (°C)	Relative Humidity (%)
**ChitPEO**	22	10	0.8	12	10	18.5	8-10
**ChitC12PEO**	22	10	0.8	23	10	18	8-12
**ChitC14PEO**	22	3 PEO + 6 ChitC14PEO	0.2	22	30	17-18	4-10
**ChitC16PEO**	22	3 PEO + 6 ChitC16PEO	0.9	23	10	18-19	4-8
